# The Integration of the Pre-Treatment Neutrophil-to-Lymphocyte Ratio in the Eighth Edition of the AJCC Staging System for Nasopharynx Cancer

**DOI:** 10.3389/fonc.2021.724467

**Published:** 2021-11-11

**Authors:** Zhong-Guo Liang, Fan Zhang, Ye Li, Ling Li, Song Qu, Fang Su, Bin-Bin Yu, Ying Guan, Lu Han, Kai-Guo Li, Xiao-Dong Zhu

**Affiliations:** ^1^ Department of Radiation Oncology, Guangxi Medical University Cancer Hospital, Cancer Institute of Guangxi Zhuang Autonomous Region, Nanning, China; ^2^ Microbiome Research Centre, St George and Sutherland Clinical School, The University of New South Wales Sydney, St George Hospital, Kogarah, NSW, Australia

**Keywords:** nasopharynx cancer (NPC), neutrophil-to-lymphocyte ratio (NLR), neoplasm staging, concurrent chemoradiotherapy (CCRT), adjuvant chemotherapy

## Abstract

**Objective:**

The present study aimed to evaluate the role of integrating the pretreatment neutrophil-to-lymphocyte ratio (NLR) into the eighth edition of the AJCC staging system for nasopharynx cancer in an endemic region.

**Methods:**

Between May 2007 and December 2012, a total of 713 cases with NPC were retrospectively analyzed. The separation ability in terms of overall survival (OS), local failure-free survival (LFFS), distant metastasis-free survival (DMFS), and failure-free survival (FFS) was evaluated. The discriminatory ability was assessed using Harrell’s concordance index (c-index). Recursive partitioning analysis (RPA) was conducted and incorporated with pretreatment NLR.

**Results:**

When integrated with NLR, the separate and discriminatory abilities for N classifications were improved in terms of OS and DMFS, but not for T categories. By using Recursive partitioning analysis, five subgroups were generated. Compared with the overall stage, the integration of NLR could not enhance the separate and discriminatory abilities. However, patients in the RPA 4 group gained significant benefits in terms of OS (HR 0.390 (95%CI 0.212-0.716), P = 0.002) and FFS (HR 0.548 (95%CI 0.314-0.958), P = 0.032) from the additional adjuvant chemotherapy after concurrent chemoradiotherapy.

**Conclusion:**

The integration of NLR into the 8^th^ edition of the AJCC staging system could enhance the separation and discriminatory abilities for N classifications, but not for T categories. In addition, patients in the RPA 4 group could benefit from the addition of adjuvant chemotherapy to concurrent chemoradiotherapy.

## Introduction

Nasopharynx carcinoma (NPC) is prevalent in South-Eastern China, Malaysia, Indonesia, Singapore, Eastern Asia, and Northern Africa, with a high incidence rate of 15-50/100,000 cases per year (1, 2). Radiotherapy in combination with chemotherapy is the main therapeutic regimen for NPC. With improvements in diagnostic imaging and radiotherapy technology and the broader application of systemic therapy, the prognosis of NPC has improved significantly (3–5).

The American Joint Committee on Cancer (AJCC) Tumor-Node-Metastasis (TNM) staging system has been widely applied to estimate curative effects and to help develop therapeutic strategies. We previously reported recommendations for updating the T and N staging systems for NPC by comparing the 2008 Chinese staging system and the 7^th^ AJCC staging system (6). Recently, the 8^th^ edition of the AJCC staging system for NPC was released and is based on large-sample clinical trials using magnetic resonance imaging (MRI) and intensity modulated radiation therapy (IMRT) technology (7).

Clinically, patients with the same TNM stage may have different prognoses, which indicates the heterogeneity among patients. Therefore, it is essential to integrate other prognostic factors into the TNM staging system. A set of studies have shown that when integrated with some biomarkers, the separation and discriminatory ability can be enhanced in several tumors, including prostate cancer (8), breast cancer (9), lymphoma (10), and seminoma (11). Pre-treatment neutrophil-lymphocyte ratio (NLR) have been proved as an useful biomarker to predict overall survival in several cancers, such as gastric cancer (12), breast cancer (13), and nasopharyngeal carcinoma (14, 15). Several studies have demonstrated that NPC patients with an elevated pre-treatment NLR had poorer survival (14, 15). The present study aimed to investigate the role of integration of the pre-treatment NLR with the eighth edition of the AJCC staging system for nasopharynx cancer in an endemic region. In addition, an accurate staging system could not only predict prognosis, but also guide clinicians in making treatment decisions. Currently, controversy exists regarding the role of adjuvant chemotherapy after concurrent chemoradiotherapy for NPC (16, 17). Therefore, we also aimed to explore whether the integration of NLR could help stratify who may benefit from the additional adjuvant chemotherapy.

## Methods and Materials

### Patients

A total of 713 patients with NPC were retrospectively analyzed between May 2007 and December 2012. Patients who met the following criteria were included: (1) differentiated or undifferentiated nonkeratinizing NPC; (2) no distant metastases upon diagnosis; (3) pretreatment evaluations, including a complete patient history, physical and neurological examinations, nasopharynx and neck MRI scans, chest X-ray or computed tomography (CT) scans, abdominal ultrasonography scans, and whole-body bone scans; and (4) use of IMRT as the radiotherapy technology. Three patients had keratinizing carcinoma, and all the other patients were diagnosed with nonkeratinizing carcinoma. The median age was 45 years old. A total of 557 patients were male, and 175 were female. Stage classifications were identified according to the 8^th^ edition of the AJCC staging system by two radiation oncologists. If discordance existed between the two radiologists, a third physician’s opinion was obtained. The patient characteristics are shown in **Table 1**. Blood routine, including the items of neutrophil and lymphocyte counts, is conducted by automatic blood analyzer before treatment (China, Shenzhen, Mindray BC6900). Considering several studies had demonstrated that NPC patients with an elevated pre-treatment NLR had poorer survival, and the median of pre-treatment NLR of 713 patients was 2.07 (range, 0.63-12.03), so it was chosen as the cut-off. The Ethics Committee of Guangxi Medical University Cancer Hospital approved the study protocol, and informed consent forms were signed by participants. The data were anonymously analyzed, and all the participants’ personal information is confidential. The research was performed in accordance with relevant guidelines and regulations.

**Table 1:** Characteristics of patients with nasopharyngeal carcinoma.

Characteristics	Number of patients (%)
Sex	
male	544 (76.3%)
female	169 (23.7%)
Age, years^†^	45 (16-86)
KPS	
70-80	274 (38.4%)
90-100	439 (61.6%)
T classification*	
T1	69 (9.7%)
T2	215 (30.2%)
T3	286 (40.1%)
T4	143 (20.1%)
N classification*	
N0	62 (8.7%)
N1	252 (35.3%)
N2	294 (41.2%)
N3	105 (14.7%)
Clinical stage*	
I	16 (2.2%)
II	139 (19.5%)
III	329 (46.1%)
IV A	229 (32.1%)
Neutrophil, k/cc^†^	4.03 (0.74-14.32)
Lymphocyte, g/L^†^	1.93 (0.43-5.57)
NLR	
≤2.07	358 (50.2%)
>2.07	355 (49.8%)
Treatment regimens	
IMRT	79 (11.1%)
CCRT	184 (25.8%)
CCRT+AC	331 (46.4%)
IC+CCRT	51 (7.2%)
IC+CCRT+AC	53 (7.4%)
IC+IMRT	8 (1.1%)
IC+IMRT+AC	7 (1.0%)

*The 8th edition American Joint Committee on Cancer staging system; ^†^The median and the range of values; NLR, Neutrophil-to-lymphocyte ratio; IMRT, Intensity modulated radiotherapy; CCRT, Concurrent chemoradiotherapy; IC, Induction chemotherapy; AC, Adjuvant chemotherapy.

### Treatment Strategies

A detailed description of IMRT has been previously published (18). The prescribed dose was 68-74 Gy applied to the primary tumor, 60-71 Gy applied to any involved cervical lymph nodes, 60-66 Gy applied to the high-risk regions, and 54-60 Gy applied to the low-risk regions in 30-32 fractions/6-7 weeks. Those with stage I disease underwent IMRT alone. For patients with stage II-IVb disease, IMRT was administered in combination with a platinum-based chemotherapy regimen.

### Follow-Up and Statistical Analysis

Follow-up was conducted from the day of diagnosis to either the day of death or the day of the last follow-up. Patients were evaluated every 3 months during the first two years, every 6 months during the next three to five years, and annually thereafter until death.

Statistical analyses were performed with SPSS software (version 16.0, SPSS Inc., Chicago, IL). The endpoints, including overall survival (OS), distant metastasis-free survival (DMFS), local failure-free survival (LFFS), and failure-free survival (FFS) were calculated using the Kaplan-Meier method, and the differences were assessed with the log-rank test. Multivariate analyses with the Cox proportional hazards model were carried out. The discriminatory performance was evaluated *via* Harrell’s concordance index (c-index) (19). The c-index was calculated using the package “rms” (20) in R version 3.5.1 (http://www.r-project.org/). To compare the c-indexes, bootstrap datasets with 1000 repetitions were performed. Recursive partitioning analysis (RPA) for OS was conducted with ordinal T- and N- categories and pre-treatment NLR to derive RPA stages objectively. The RPA algorithm is based on the optimized binary partition of T- or N- categories or NLR, which would result in subgroups with relatively homogeneous survival performance. All P-values were two-sided, and P ≤ 0.05 was considered statistically significant.

## Results

**Figure 1:**
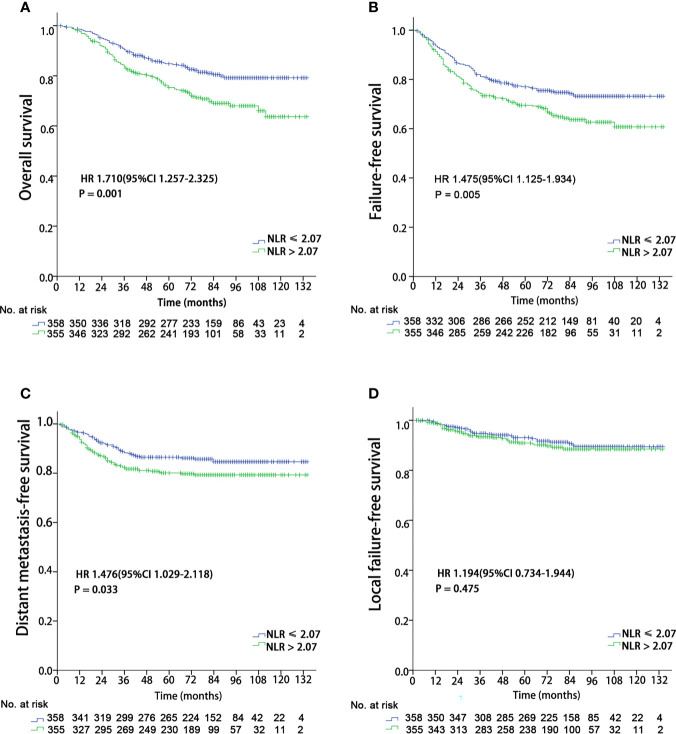
Kaplan–Meier survival curves for 713 patients stratified by the cutoff of NLR. NLR, Neutrophil-to-lymphocyte ratio. **(A)** Overall survival; **(B)** Failure-free survival; **(C)** Distant metastasis failure-free survival; **(D)** Local failure-free survival.

With a median follow-up of 77 months (range, 2-134 months), a total of 171 (24%) patients died, 121 (17%) developed distant metastasis, and 65 (9.1%) developed local recurrence. Univariate analysis showed that patients with a pretreatment NLR > 2.07 had poor OS (HR 1.710 (95%CI 1.257-2.325), P = 0.001), DMFS (HR 1.476 (95%CI 1.029-2.118), P = 0.033), and FFS (HR 1.475 (95%CI 1.125-1.934), P = 0.005) than those with a NLR ≤ 2.07, while no significant difference was found in LFFS (HR 1.194 (95%CI 0.734-2.118), P = 0.475) (**Figure 1**). The univariate analysis also indicated that sex, age (continuous), and T and N classifications were significant prognostic factors for OS. The multivariate analysis revealed that age (continuous), pre-treatment NLR, and T and N classifications were significant factors for OS (details are shown in **Table 2**).

**Table 2:** Univariate and multivariate analyses for the overall survival using Cox regression model.

Endpoints	Univariate analysis	P	Multivariate analysis	P
	HR (95%CI)		HR (95%CI)	
Sex				
Female *vs* male	0.680 (0.461-1.004)	0.05	0.753 (0.508-1.115)	0.156
Age				
Continuous	1.042 (1.029-1.056)	<0.001	1.047 (1.033-1.062)	<0.001
Chemotherapy				
Yes *vs* No	0.976 (0.612-1.558)	0.919		
T classification				
T3-4 *vs* T1-2	2.490 (1.751-3.540)	<0.001	2.036 (1.422-2.915)	<0.001
N classification				
N2-3 *vs* N0-1	1.849 (1.342-2.547)	<0.001	1.941 (1.398-2.695)	<0.001
NLR				
>2.07 *vs* ≤2.07	1.710 (1.257-2.325)	0.001	1.593 (1.166-2.177)	0.003

HR, Hazard Ratio; NLR, Neutrophil-to-lymphocyte ratio.

### T Classification

**Figure 2:**
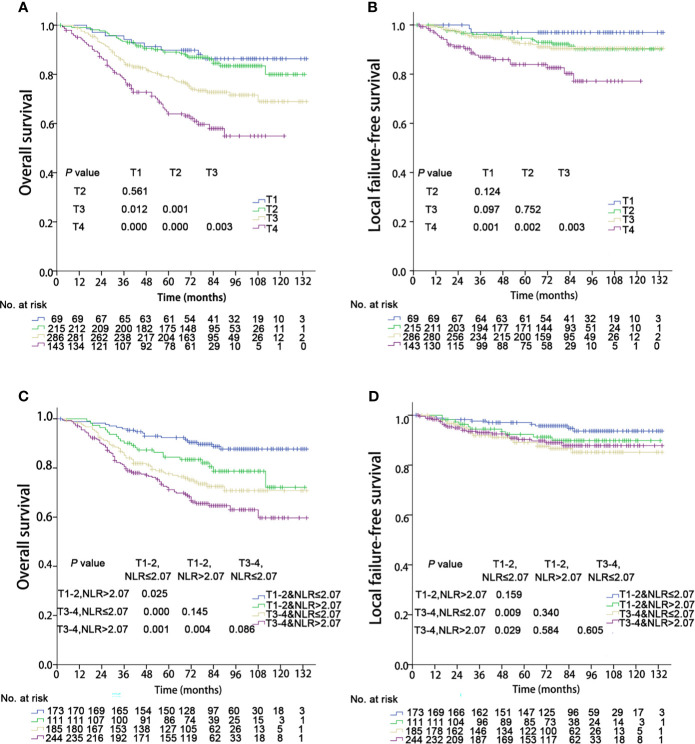
Kaplan–Meier survival curves of 713 patients stratified by the T and T & NLR classifications. NLR, Neutrophil-to-lymphocyte ratio. **(A, C)** Overall survival; **(B, D)** Local failure-free survival.

The OS and LFFS curves for the T categories are shown in **Figure 2**. Based on the 8^th^ edition staging system, there were significant differences in OS between the T subgroups, except in the comparison of the T1 and T2 classifications (details are shown in **Figure 2A**). However, there only existed a significant difference in LFFS between T4 and the other T categories (P < 0.05; **Figure 2B**). When integrated with NLR, a significant difference in OS was found between “T1-2 & NLR ≤ 2.07” and “T1-2 & NLR > 2.07”, but no significant differences existed between “T1-2 & NLR > 2.07” and “T3-4 & NLR ≤ 2.07”, “T3-4 & NLR ≤ 2.07” and “T3-4 & NLR > 2.07” (**Figure 2C**). Regarding LFFS, significant differences were only observed between “T1-2 & NLR ≤ 2.07” and “T3-4 & NLR ≤ 2.07”, “T1-2 & NLR ≤ 2.07” and “T3-4 & NLR > 2.07” (**Figure 2D**). After integration of NLR, the C-index became smaller for both OS and LFFS (details are shown in **Table 3**), which meant that the discriminatory ability was not improved.

**Table 3:** Univariate analysis for T and N classifications associated with overall survival, local failure-free survival, and distant metastasis free survival.

	HR (95%CI)	P	C-index		HR (95%CI)	P	C-index	P*
OS				OS				
T1	reference			T1-2&NLR ≤ 2.07	reference			<0.001
T2	1.231 (0.587-2.579)	0.583	0.632	T1-2&NLR>2.07	1.947 (1.054-3.598)	0.033	0.628	
T3	2.409 (1.205-4.815)	0.013	(0.590-0.674)	T3-4&NLR ≤ 2.07	2.803 (1.647-4.772)	<0.001	(0.585-0.671)	
T4	4.119 (2.031-8.353)	<0.001		T3-4&NLR>2.07	3.826 (2.320-6.308)	<0.001		
								
LFFS				LFFS	0.088			
T1	reference			T1-2&NLR ≤ 2.07	reference			<0.001
T2	2.956 (0.683-12.798)	0.147	0.626	T1-2&NLR>2.07	1.859 (0.755-4.577)	0.177	0.580	
T3	3.259 (0.768-13.832)	0.109	(0.558-0.694)	T3-4&NLR ≤ 2.07	2.666 (1.227-5.796)	0.013	(0.511-0.649)	
T4	7.680 (1.808-32.629)	0.006		T3-4&NLR>2.07	2.299 (1.067-4.953)	0.034		
								
								
OS				OS				
N0	reference			N0-1&NLR ≤ 2.07	reference			<0.001
N1	1.245 (0.628-2.468)	0.531	0.593	N0-1&NLR>2.07	2.104 (1.225-3.614)	0.007	0.610	
N2	2.002 (1.035-3.875)	0.039	(0.552-0.635)	N2-3&NLR ≤ 2.07	2.229 (1.329-3.737)	0.002	(0.567-0.653)	
N3	2.810 (1.395-5.663)	0.004		N2-3&NLR>2.07	3.268 (2.003-5.332)	<0.001		
								
DMFS				DMFS				
N0	reference			N0-1&NLR ≤ 2.07	reference			<0.001
N1	1.551 (0.538-4.472)	0.416	0.640	N0-1&NLR>2.07	1.238 (0.590-2.596)	0.573	0.644	
N2	3.683 (1.339-10.131)	0.012	(0.591-0.689)	N2-3&NLR ≤ 2.07	2.611 (1.411-4.830)	0.002	(0.593-0.694)	
N3	5.549 (1.962-15.697)	0.001		N2-3&NLR>2.07	3.770 (2.098-6.775)	<0.001		

P* < 0.05 was considered statistically significant difference between the two c-indexes. HR, Hazard Ratio; NLR, Neutrophil-to-lymphocyte ratio; OS, Overall survival; LFFS, Local failure-free survival; DMFS, Distant metastasis-free survival.

### N Classification

**Figure 3:**
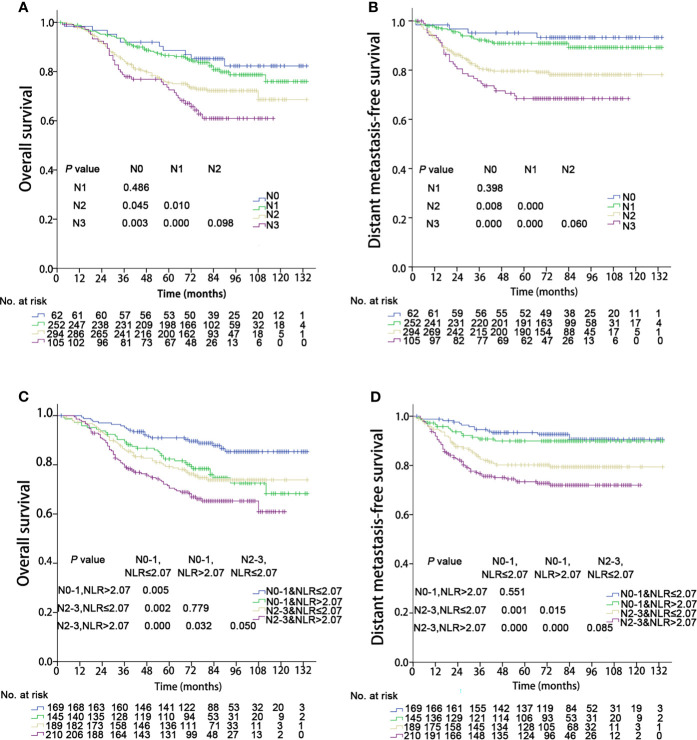
Kaplan–Meier survival curves of 713 patients stratified by the N and N & NLR classifications. NLR, Neutrophil-to-lymphocyte ratio. **(A, C)** Overall survival; **(B, D)** Distant metastasis failure-free survival.

The OS and DMFS curves for the N subsets are shown in **Figure 3**. Significant differences were found between the N subsets in terms of OS and DMFS for the 8^th^ edition AJCC staging system, except for comparison of the N0 and N1 as well as N2 and N3 classifications (details are shown in **Figures 3A, B**). After integration of NLR, there were significant differences in OS between the updated subgroups except for the comparison of “N0-1 & NLR > 2.07” and “N2-3 & NLR ≤ 2.07” (**Figure 3C**). In terms of DMFS, there were still no significant differences between the “N0-1 & NLR ≤ 2.07” and “N0-1 & NLR > 2.07” subgroups and between the “N2-3 & NLR ≤ 2.07” and “N2-3 & NLR > 2.07” subgroups. Cox multivariate regression analysis showed that the N classifications were independent prognostic indicators of DMFS and OS in the two staging systems (P< 0.001). When integrated with NLR, significant improvements in the C-index of OS and DMFS were observed (details are shown in **Table 3**).

### Recursive Partitioning Analysis

**Figure 4:**
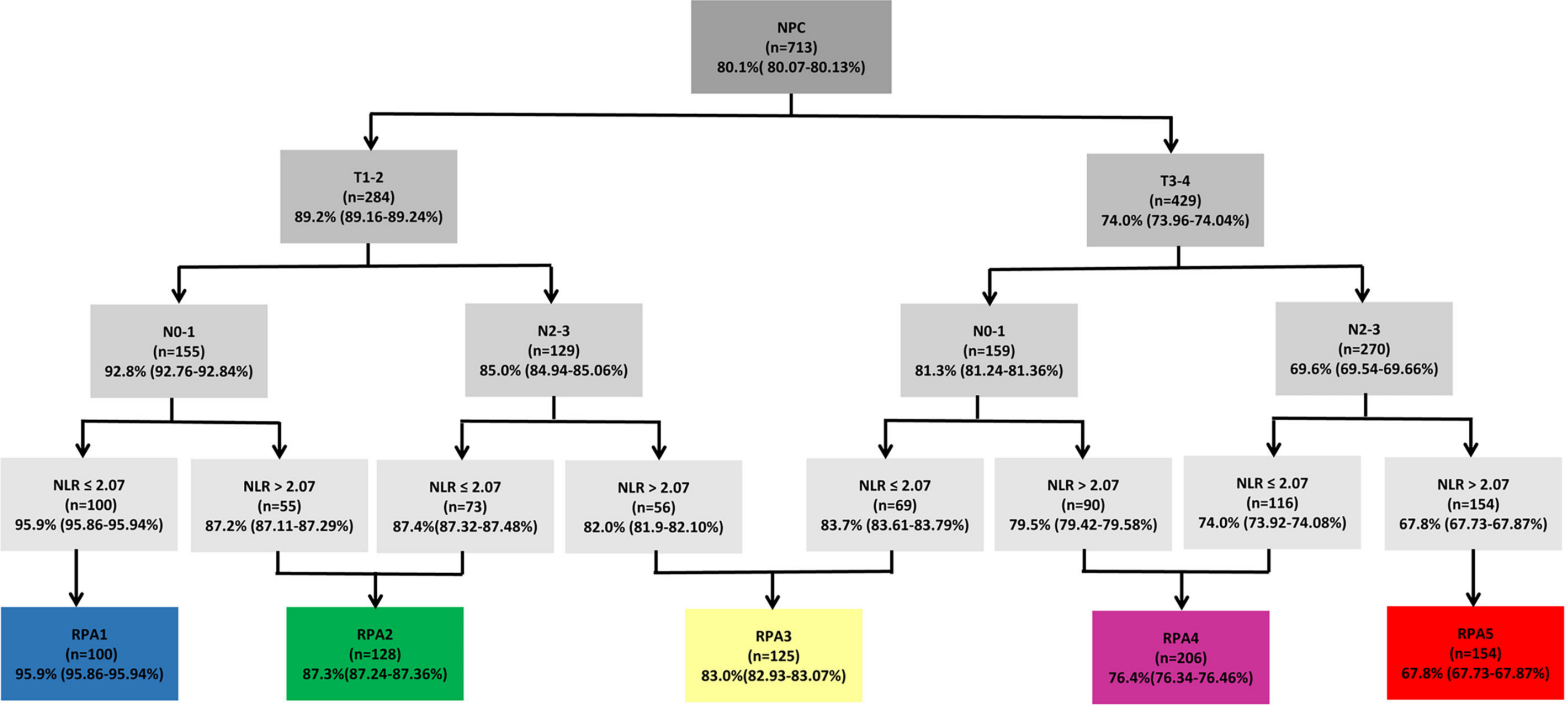
Recursive partitioning analysis for the endpoint of 5-year overall survival of 713 patients, based on the optimized binary partition of T-, N- categories and NLR. RPA, Recursive partitioning analysis; NLR, Neutrophil-to-lymphocyte ratio.

**Figure 5:**
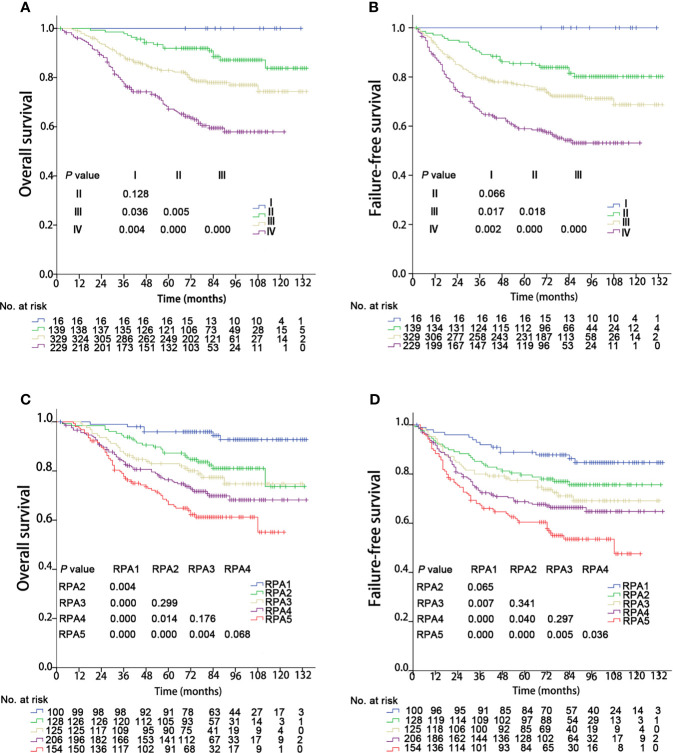
Kaplan–Meier survival curves of 713 patients stratified by the overall stage and RPA groups. RPA, Recursive partitioning analysis. **(A, C)** Overall survival; **(B, D)** Failure-free survival.

Recursive partitioning analysis classified NPC patients into eight categories with disparate outcomes for OS (**Figure 4**). Then 5-year overall survival rates in each group were calculated by using the Kaplan-Meier method, with 95.9%, 87.2%, 87.4%, 82.0%, 83.7%, 79.5%, 74.0%, and 67.8% in eight subgroups, respectively. Those with 5-year OS > 90% were classified as the best prognosis group (RPA1), and those with 5-year OS ≤70% were classified as the poor prognosis group (RPA5). Those with 5-year OS > 70%, and ≤80% were identified the intermediate prognosis group (RPA4). Those with 5-year OS > 80%, and ≤90% were identified as the good prognosis group. The good prognosis group consisted of four subgroups, and the 5-year rates of OS were 87.2%, 87.4%, 82.0%, and 83.7%, respectively. Then the good prognosis group was divided into two groups. The two subgroups with 5-year OS of 87.2% and 87.4% were merged into RPA2, and the other two subgroups with 5-year OS of 82.0%, and 83.7% were merged into RPA3 (**Figure 4**). Five-year OS was significantly different between RPA groupings (RPA1 to RPA5: 95.9%, 87.3%, 83.0%, 76.4%, and 67.8%, respectively). **Figure 5** shows the FFS and OS curves for the overall stage and RPA groups. According to the 8^th^ AJCC staging system, significant differences were found between the clinical stages in terms of OS and FFS, except for comparison of stages I and II (details are shown in **Figures 5A, B**). When using the RPA stages, patients with RPA1 stage had better OS than those with RPA2 stage, but no significant differences were observed between patients with the RPA2 and RPA3 stages, between the RPA3 and RPA4 stages, and between the RPA4 and RPA5 stages (**Figure 5C**). Regarding FFS, significant differences were found between the subgroups, except for the comparisons of the RPA1 and RPA2, RPA2 and RPA3, and RPA3 and RPA4 groups (**Figure 5D**). The c-indexes of OS and FFS were not improved when using RPA classifications (details are shown in **Table 4**).

**Table 4:** Univariate analysis for the overall stage and RPA classes associated with overall survival, and failure-free survival.

	HR (95%CI)	P	C-index		HR (95%CI)	P	C-index	P*
OS				OS				
I-II	reference		0.646	RPA 1	reference		0.646	0.863
III	2.396 (1.391-4.128)	0.002	(0.605 - 0.687)	RPA 2	3.268 (1.330-8.032)	0.010	(0.603-0.690)	
IV	4.893 (2.862-8.367)	<0.001		RPA 3	4.337 (1.789-10.516)	0.001		
				RPA 4	5.939 (2.560-13.779)	<0.001		
				RPA 5	8.424 (3.626-19.571)	<0.001		
								
FFS				FFS				
I-II	reference		0.619	RPA 1	reference		0.611	<0.001
III	1.906 (1.223-2.970)	0.004	(0.583 - 0.656)	RPA 2	1.798 (0.953-3.391)	0.070	(0.572- 0.649)	
IV	3.509 (2.262-5.445)	<0.001		RPA 3	2.280 (1.226-4.240)	0.009		
				RPA 4	2.834 (1.594-5.040)	<0.001		
				RPA 5	4.066 (2.285-7.235)	<0.001		

RPA, Recursive partitioning analysis; P* < 0.05 was considered statistically significant difference between the two c-indexes; HR, Hazard Ratio; NLR, Neutrophil-to-lymphocyte ratio; OS, Overall survival; FFS, Failure-free survival.

**Figure 6:**
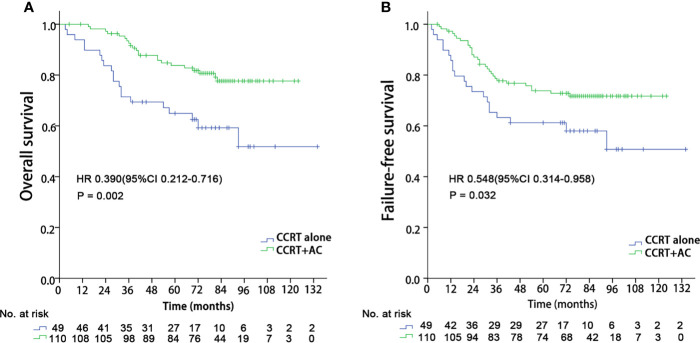
Kaplan–Meier survival curves in the CCRT + AC and CCRT alone arms for patients in the RPA 4 group. CCRT, Concurrent chemoradiotherapy; AC, Adjuvant chemotherapy; RPA, Recursive partitioning analysis; **(A)** Overall survival; **(B)** Failure-free survival.

We performed subgroup analyses to explore whether the integration of NLR could help identify who may benefit from the additional adjuvant chemotherapy by comparing concurrent chemoradiotherapy followed by adjuvant chemotherapy *versus* concurrent chemoradiotherapy alone. When using the 8^th^ edition AJCC staging system, no significant benefits of adjuvant chemotherapy were found in any stage groups. For those 206 patients in RPA4, 77.2% (159/206) patients received concurrent chemoradiotherapy alone or concurrent chemoradiotherapy followed by adjuvant chemotherapy. We observed that patients in the RPA 4 group gained significant OS benefits (HR 0.390 (95%CI 0.212-0.716), P = 0.002) and FFS (HR 0.548 (95%CI 0.314-0.958), P = 0.032) from the additional adjuvant chemotherapy (**Figure 6**). However, no significant survival benefits were found for the additional adjuvant chemotherapy to concurrent chemoradiotherapy for other RPA groups. Meanwhile, the value of induction chemotherapy was also investigated. A total of 119 patients (16.7%) received induction chemotherapy, with 9 in RPA 1, 17 in RPA 2, 20 in RPA 3, 34 in RPA 4, and 39 in RPA 5, respectively. Then we compared the efficacy of the treatment regimens with or without induction chemotherapy in RPA 4 and 5 groups. It was found that no survival benefits were gained from induction chemotherapy in both RPA 4 and 5 groups.

## Discussion

In the present study, we explored the role of integrating pre-treatment NLR with the 8^th^ edition AJCC staging system. We observed that the integration of NLR could enhance the separate and discriminatory abilities for the N category, but not for the T classification. In addition, it could help identify who may benefit from the additional adjuvant chemotherapy after concurrent chemoradiotherapy.

In a meta-analysis involving 7,031 patients, an increased NLR was related to a poor OS and PFS (PFS) (21), which was consistent with the present study. In another two retrospective studies, Liao et al. and Ye et al. also observed that patients with a high NLR had significantly lower PFS and OS (15, 22). What is the potential mechanism by which NLR can affect tumor prognosis? As is well known, the tumor microenvironment (TME) plays a key role in tumorigenesis, proliferation, invasion, and metastasis. As inflammatory markers, neutrophils and lymphocytes are both important components of TME (23, 24). Neutrophils can activate tumor initiation by inducing the formation of reactive oxygen species (ROS), reactive nitrogen species (RNS) and proteases, as well as promoting tumor proliferation (23). In addition, neutrophils can also motivate metastasis formation *via* inhibiting natural killer function and facilitating the extravasation of tumor cells through the secretion of IL1β and matrix metalloproteinases (25). Several studies have reported the potential mechanism why neutrophil correlate with poor prognosis of cancer patients. Wang et al. found that patients with gastric cancer showed a significantly higher neutrophil infiltration in tumors, and the tumor-activated neutrophils fostered immune suppression and disease progression through granulocyte-macrophage colony-stimulating factor-PD-L1 (GM-CSF-PD-L1) pathway (26). In 2021, Kajioka et al. reported that neutrophil extracellular traps (NETs) induced the epithelial to mesenchymal transition in pancreatic cancer cells and thereby promoted their migration and invasion (27). Lymphocyte, including cytotoxic T cells, Th1 helper cells and B cells, can orchestrate tumor cell elimination (24). Therefore, an abundance of lymphocytes may result in poor prognoses. That is to say if patients have higher level of neutrophil and lower level of lymphocyte, their prognoses may be poor.

The peripheral NLR may become an easily measured, cost-effective and reproducible marker associated with clinical practice. However, there still exist some issues that need to be resolved. For example, the cutoff of NLR is not unique in different research institutes, ranging from 2.28 to 5.0 according to the published results (21). It is also unclear whether the level of NLR can be altered to improve prognosis by targeted treatment. Therefore, more studies need to be performed to assess the widespread use of this biomarker.

In the era of precision medicine, heterogeneities among patients require oncologists to integrate other prognostic factors into the TNM staging system. For example, prostatic specific antigen (PSA), as a powerful biomarker for prostate cancer, has been merged into the AJCC staging system to help divide patients into different risk groups (8). The expression levels of estrogen receptors (ER) and progesterone receptors (PR), as well as Her-2 for breast cancer, guide oncologists in predicting prognosis and developing the corresponding therapeutic strategies (9). Our study showed that the integration of NLR could help improve the separation and discriminatory abilities for the N category, but not for T classification. It is urgent to conduct multicenter studies to identify the role of integrating NLR with the TNM staging system for NPC.

Currently, the role of adjuvant chemotherapy (AC) after concurrent chemoradiotherapy (CCRT) for NPC is still unclear. A multicenter randomized controlled trial and a retrospective study with 2,263 patients both showed that the addition of adjuvant chemotherapy to CCRT could not provide significant survival benefits (16, 28). However, a multi-institutional retrospective study with 380 patients in the CCRT alone arm and 327 patients in the CCRT-AC arm revealed that AC can significantly improve survival (17). In the present study, we observed that patients in the group RPA 4 (T3-4N0-1&NLR>2.07, or T3-4N2-3&NLR ≤ 2.07) may gain a survival benefit from the addition of adjuvant chemotherapy to concurrent chemoradiotherapy. However, the samples in both arms were not large. It is essential to conduct prospective trials with large samples to verify the results.

There are some limitations of the present study. First, as a retrospective study, selection bias may have occurred because patients were included only if they met specific selection criteria. However, these findings can help us design and conduct prospective studies to investigate the truth. Second, the sample size was not large. Third, the patients were from a single hospital, which may result in selection bias. Finally, a set of studies has shown that the presence of Epstein-Barr viral DNA (EBV DNA) in plasma (29) has a significant influence on the prognosis of NPC patients. However, these relevant data are not available for all patients, and plasma EBV DNA assay by RT-PCR (reverse transcription-polymerase chain reaction) is not yet a routine investigation in many centres, especially in low income countries and cities. Additionally, most published studies are based on laboratory-derived test of the individual institute: the lowest detection limit varies widely among different institutions, resulting in variation in false-negative rates and recommended cutoff values (30). Therefore, considering that the updated stage system should have wide popularization and application prospects, we did not include this factor in the analysis.

In summary, the integration of NLR into the 8^th^ edition of the AJCC staging system could significantly improve the separation and discriminatory abilities for N classification, but not for T category. Additionally, it could help stratify patients who may gain a survival benefit from the addition of adjuvant chemotherapy to concurrent chemoradiotherapy. More prospective trials with large samples are essential to verify these results.

## Data Availability Statement

The original contributions presented in the study are included in the article/supplementary material. Further inquiries can be directed to the corresponding author.

## Ethics Statement

The studies involving human participants were reviewed and approved by The Ethics Committee of Guangxi Medical University Cancer Hospital. Written informed consent to participate in this study was provided by the participants’ legal guardian/next of kin.

## Author Contributions

X-DZ contributed to conception and design of the study. Z-GL, FZ, K-GL, LH, and FS organized the database. Z-GL and FZ performed the statistical analysis. Z-GL, FZ, and YL wrote the first draft of the manuscript. LL, SQ, B-BY, and YG wrote sections of the manuscript. All authors contributed to manuscript revision, read, and approved the submitted version.

## Funding

This work was sponsored by grants from the National Natural Science Foundation of China (No. 81760544), Key R&D Program of Guangxi (AB18221007), Project of Guangxi Medical and Health Appropriate Technology Development and Extension Application (No. S2018001), Guangxi Natural Science Foundation (No. 2020GXNSFBA159002), the Health Commission of Guangxi Zhuang Autonomous Region (No.Z20200333). The funders had no role in study design, data collection and analysis, decision to publish, or preparation of the manuscript.

## Conflict of Interest

The authors declare that the research was conducted in the absence of any commercial or financial relationships that could be construed as a potential conflict of interest.

## Publisher’s Note

All claims expressed in this article are solely those of the authors and do not necessarily represent those of their affiliated organizations, or those of the publisher, the editors and the reviewers. Any product that may be evaluated in this article, or claim that may be made by its manufacturer, is not guaranteed or endorsed by the publisher.
